# Identification and Integration of LRG1-Induced Differentially Expressed Gene (DEG) Hub Profiles in Breast Cancer Cells

**DOI:** 10.3390/ijms27083613

**Published:** 2026-04-18

**Authors:** Federico Osorio-Antonio, Daniela Michel Diaz-González, Gabriela Elizabeth Campos-Viguri, José Manuel Sánchez-López, José Luis Cortez-Sánchez, Francisco Castelán, Jesús Ramses Chávez-Rios, Paola Maycotte-González, Paulina Cortés-Hernández, Oscar Peralta-Zaragoza, Elizabeth Bautista-Rodríguez

**Affiliations:** 1 Departamento de Genética Médica, Hospital Infantil de Tlaxcala, Servicios de Salud del Instituto Mexicano del Seguro Social para el Bienestar, Tlaxcala 90606, Mexico; federico.osorio@upaep.edu.mx (F.O.-A.); cienciaflosan@gmail.com (J.M.S.-L.); 2 Facultad de Biotecnología, Universidad Popular Autónoma del Estado de Puebla, Puebla 72410, Mexico; 3 Departamento de Fisiología, Biofísica y Neurociencias, Centro de Investigación y de Estudios Avanzados del Instituto Politécnico Nacional, Ciudad de México 07360, Mexico; danielamichel.diaz@upaep.edu.mx; 4 Centro de Investigación en Ciencia Aplicada y Tecnología Avanzada Unidad Morelos, Instituto Politécnico Nacional, Atlacholoaya 62790, Mexico; gaby_gecv@hotmail.com; 5 Dirección de Infecciones Crónicas y Cáncer, Centro de Investigación Sobre Enfermedades Infecciosas, Instituto Nacional de Salud Pública, Cuernavaca 62100, Mexico; 6 Environmental Epigenetics and Mental Health Laboratory, Faculty of Chemical Biological Sciences, Autonomous University of Campeche, Campeche 24085, Mexico; jose.cortez@udlap.mx; 7 Health Sciences, University of the Americas Puebla (UDLAP), Puebla 72810, Mexico; 8 Departamento de Biología Celular y Fisiología, Unidad Foránea Tlaxcala, Instituto de Investigaciones Biomédicas, Universidad Nacional Autónoma de México, Tlaxcala 90070, Mexico; fcocastelan@iibiomedicas.unam.mx (F.C.); jchavez_rios@iibiomedicas.unam.mx (J.R.C.-R.); 9 Centro Tlaxcala de Biología de la Conducta, Universidad Autónoma de Tlaxcala, Tlaxcala 90070, Mexico; 10 Centro de Investigación Biomédica de Oriente, Instituto Mexicano del Seguro Social, Puebla 74360, Mexico; bisbenzimida@gmail.com (P.M.-G.); paulina.cortes.hernandez@gmail.com (P.C.-H.); 11 Multidisciplinary Laboratory in Biomedicine, Biotechnology and Integrative Bioinformatics Applied to Health (LAMB3IS), Faculty of Health Sciences, Autonomous University of Tlaxcala (UATx), Zacatelco 90750, Mexico; 12 Institut de Pharmacologie et de Biologie Structurale (IPBS-CNRS), 31077 Toulouse, France

**Keywords:** breast cancer, DEGs, LRG1, MAPK, PI3K, RAS

## Abstract

Breast carcinoma is a major cause of cancer-related mortality among women worldwide. Identifying novel molecular targets remains essential, particularly for aggressive triple-negative breast cancer (TNBC). Leucine-rich alpha-2-glycoprotein 1 (LRG1) has been linked to tumor progression and angiogenesis, but its molecular mechanisms in breast cancer are poorly defined. We evaluated the effects of recombinant human LRG1 (rhLRG1) on cell viability and migration in MDA-MB-231 TNBC cells and performed transcriptomic profiling followed by functional enrichment analyses using GenArise, Cytoscape, and R-based tools. RhLRG1 treatment significantly increased cell viability and migration. Transcriptomic analysis revealed activation of key oncogenic cascades, including the PI3K/AKT, MAPK, and RAS signaling pathways. Hub-gene analysis identified upregulated genes involved in proliferation (*NRAS*, *STAT5B*, *IGF2*), angiogenesis (*PGF*, *ANGPT2*), and apoptosis (*CASP8*, *BAD*), whereas downregulated genes were associated with apoptotic resistance (*BCL2*, *MCL1*) and adhesion (*LAMB1*, *ITGB4*). Functional enrichment highlighted LRG1’s role in the bioinformatic analysis of differentially expressed genes that were obtained from microarray assays. LRG1 remodels the tumor microenvironment by promoting proliferation, angiogenesis, and apoptotic sensitivity while repressing resistance-related genes. These findings position LRG1 as a potential diagnostic biomarker and therapeutic target for advanced breast carcinoma.

## 1. Introduction

Breast carcinoma remains the most frequently diagnosed malignancy among women worldwide and constitutes the second leading cause of cancer-related mortality in this population [1]. Within the heterogeneous spectrum of breast carcinomas, triple-negative breast cancer (TNBC) represents a distinct and highly aggressive subtype [2,3,4]. TNBC is defined by the absence of the estrogen receptor (ER), progesterone receptor (PR), and human epidermal growth factor receptor 2 (HER2) [5]. This phenotype is characterized by marked biological aggressiveness and poor clinical prognosis, accounting for approximately 10–15% of all breast cancer cases [5]. The intrinsic heterogeneity of TNBC is closely associated with an increased risk of local recurrence and distant metastasis [6]. The lack of ER, PR, and HER2 therapeutic targets renders TNBC refractory to conventional hormonal or targeted therapies [7,8,9,10]. Consequently, despite significant advances—such as those derived from single-cell transcriptomic profiling of the tumor immune microenvironment—the clinical management of TNBC remains exceptionally challenging, reinforcing the urgent need for novel therapeutic strategies that can overcome chemoresistance [9,10]. Despite extensive research into the mechanisms of cancer progression and resistance, the pathophysiology of TNBC remains largely undefined [11,12,13].

The LRG1 protein, encoded by the *LRG1* gene located on chromosome 19p13.3 [14], colocalizes with several genes encoding neutrophil granule enzymes, linking its expression to granulocyte differentiation [14]. Structurally, LRG1 belongs to the leucine-rich repeat (LRR) protein family [15]. LRR proteins participate in diverse biological processes, including protein–protein interactions, signal transduction, cell adhesion, proliferation, migration, development, apoptosis, immune response, angiogenesis, and tissue remodeling [15]. LRG1 is a secreted protein and can therefore be detected as a circulating biomarker. Overexpression of LRG1 has been consistently associated with poor prognosis and advanced disease progression across multiple cancers, including gastric [16], prostate [17], ovarian [18], colorectal [19,20], endometrial [21], and breast cancer [22,23]. In breast cancer, high LRG1 levels correlate strongly with aggressive disease phenotypes. Moreover, patients with high LRG1 expression exhibit significantly reduced disease-free and overall survival [22,23]. In addition, LRG1 expression has been associated with pathological indicators of systemic dissemination, lymph node involvement, and distant metastasis, particularly in HER2-positive and TNBC subtypes [22,23]. Collectively, these findings position LRG1 as a key molecular determinant associated with invasive potential and poor prognosis in breast carcinoma.

Extensive research has approached the regulatory mechanisms controlling LRG1 expression and its downstream signaling pathways in diverse cancers, including pancreatic [24], gastric [25], colorectal [26], lung [27], and bladder malignancies [28]. A major discovery has been the involvement of pro-inflammatory cytokines inducing LRG1 transcription. Specifically, IL-6 and IL-22 activate the transcription factor STAT3, which subsequently upregulates LRG1 expression [29,30]. In parallel, IL-1 and TNF-α activate the NF-κB pathway, further enhancing LRG1 transcription [29,30]. This dual regulation is supported by the presence of STAT3 and NF-κB response elements within the LRG1 promoter region [29,30]. Beyond transcriptional regulation, LRG1 activates several oncogenic pathways, notably TGF-β1 signaling. LRG1-induced activation of TGF-β1 engages SMAD1, SMAD5, and SMAD8 transcription factors, ultimately promoting angiogenesis [31,32]. This is particularly relevant in breast cancer, where TGF-β1 exerts context-dependent effects—acting as a tumor suppressor in the early stages but switching to a pro-oncogenic driver in advanced disease. High levels of TGF-β1 promote epithelial–mesenchymal transition (EMT), facilitating migration, invasion, and metastasis [31,32]. LRG1 also enhances angiogenesis through HIF-1α–mediated upregulation of VEGFA [20]. Despite these insights, the molecular mechanisms and downstream signaling networks regulated by LRG1, especially in TNBC, remain incompletely understood. Therefore, a comprehensive analysis of hub differentially expressed genes (DEGs) induced by LRG1 stimulation is proposed as a valuable strategy to delineate the signaling pathways in which LRG1 participates, thereby clarifying its functional role in TNBC biology.

In this study, we employed the MDA-MB-231 cell line as an in vitro model, given its phenotypic similarity to aggressive TNBC and its high migratory capacity [33]. Our primary objective was to determine whether recombinant human LRG1 (rhLRG1) directly influences key oncogenic processes, specifically cell viability and migration. In addition, we performed transcriptomic analysis to approach the activation of major intracellular cascades, including PI3K, MAPK, and RAS pathways, following LRG1 stimulation. A functional correlation, based on Gene Ontology (GO) and Kyoto Encyclopedia of Genes and Genomes (KEGG) enrichment analyses, was approached for hub DEGs, and the resulting data were integrated into a network constructed in Cytoscape 3.10.3 using the ClueGO (West Midlands, UK) and CluePedia plugins (West Midlands, UK).

## 2. Results

### 2.1. LRG1 Increases Viability in Breast Cancer Cells

**Figure 1:**
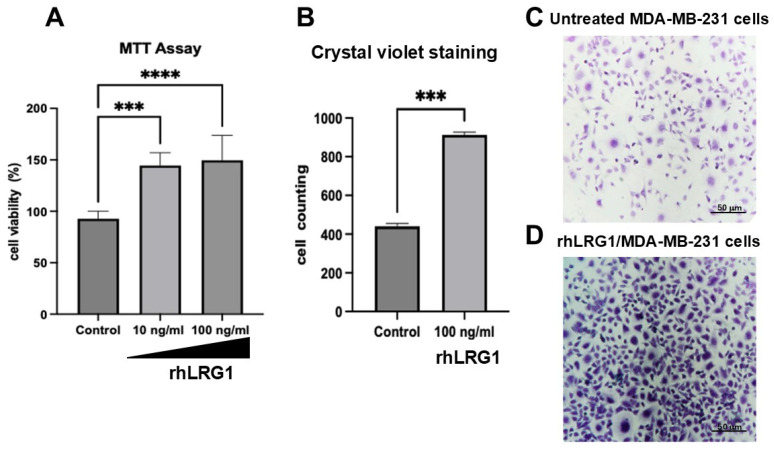
LRG1 increases proliferation of MDA-MB-231 cells. (**A**) Cellular viability was assayed by MTT in untreated (control) and hrLRG1-treated cells. Representative microphotographs were acquired using a Vista Vision inverted microscope with a 100× objective of crystal violet-stained cells treated or not ((**B**), control) with 100 ng/mL LRG1 (**C**). (**D**) Cell density is estimated by counting crystal violet-stained cells (control and rhLRG1 cells). Data are means ± SD. ***, *p* < 0.001; ****, *p* < 0.0001.

Despite limited reports proposing a protective role in specific contexts, a substantial and consistent body of evidence characterizes LRG1 as a significant biomarker for the diagnosis and clinical assessment of numerous cancer types [16,17,18,19,20,21,22,23]. To investigate LRG1’s influence on cellular proliferation and viability, the MTT assay was performed on MDA-MB-231 cells, a highly aggressive TNBC subtype model. Cells were treated with rhLRG1 and analyzed at 0 h, 3 h, 6 h, and 24 h post-treatment. The results demonstrated that both 10 and 100 ng/mL rhLRG1 significantly increased (*p* < 0.001, *p* < 0.0001) the viability of MDA-MB-231 cells, as compared to untreated cells (Figure 1A). Furthermore, data from crystal violet staining revealed an evident increase in cell density following 100 ng/mL hrLRG1 (Figure 1B,C). Quantitative cell counting corroborated this observation, showing a significant augmentation (*p* < 0.001) in stained cells in response to the rhLRG1 treatment (Figure 1D). This evidence indicates that LRG1 enhances the viability and proliferation of MDA-MB-231 cells. This finding underscores the growing interest in LRG1, not only regarding its clinical utility as a biomarker but also regarding understanding the molecular mechanisms through which it actively contributes to the process of carcinogenesis.

### 2.2. LRG1 Induces Alteration in Cell Migration in Breast Cancer Cells

**Figure 2:**
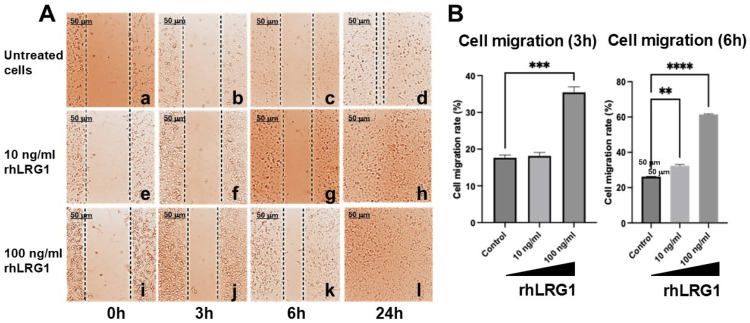
LRG1 enhances migration of MDA-MB-231 cells. Cellular migration was evaluated by wound-healing assays. (**A**) Representative microphotographs were acquired using a Vista Vision inverted microscope with a 100× objective, captured at 0 h, 3 h, 6 h, and 24 h after making the stria in untreated (**a**–**d**) and hrLRG1-treated cells (10 ng/mL, (**e**–**h**); 100 ng/mL, (**i**–**l**)). (**B**) Cell migration as a percentage (%) was measured 3 and 6 h post-rhLRG1 treatments. Data are means ± SD. **, *p* < 0.01; ***, *p* < 0.001; ****, *p* < 0.0001.

Prior investigations concerning human colorectal cancer, pancreatic adenocarcinoma, and glioma have consistently demonstrated a positive correlation between elevated LRG1 protein levels and enhanced cellular migration [20,24,34]. To ascertain whether LRG1 exerts similar effects on the motility of breast cancer cells, functional assays were performed to evaluate its influence on the migration of MDA-MB-231 cells by using wound-healing assays. As compared to untreated cells (Figure 2A, lanes a–d), 10 ng/mL and 100 ng/mL rhLRG1 increased the migration of MDA-MB-231 cells after a 24 h incubation period (Figure 2A, lanes e–h, i–l). Further statistical analyses showed that 100 ng/mL hrLRG1 was able to increase the migration rate of MDA-MB-231 cells as early as 3 h post-treatment (*p* < 0.001; Figure 2B), while 10 ng/mL rhLRG1 favored cell migration as measured 6 h post-treatment (*p* < 0.001; Figure 2B right). By 24 h post-treatment, both rhLRG1 doses reached approximately 100% wound coverage (Figure 2A, lanes h and l). These findings support the notion that LRG1 facilitates the reestablishment of cellular motility, thereby modulating cell migration within the context of breast cancer progression. Thus, this particular pattern of cellular motility is often characteristic of cells possessing the capacity to lyse the extracellular matrix (ECM) and navigate the surrounding interstitial spaces.

### 2.3. Identification of Hub Differentially Expressed Gene (DEG) Profiles from Heatmap of Breast Cancer Cells Treated with LRG1

**Figure 3:**
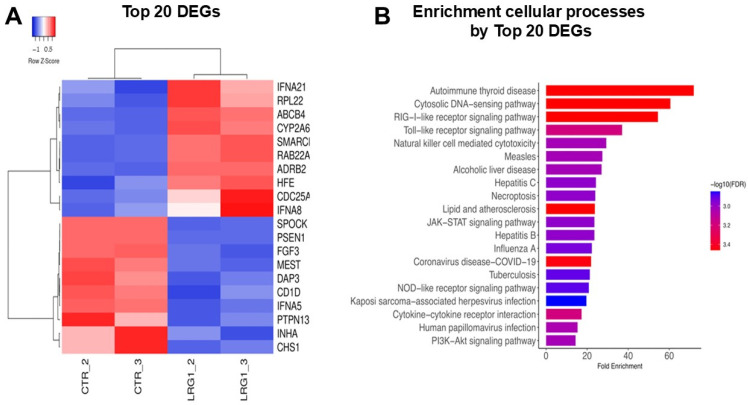
Global heatmap of top 20 DEGs and enrichment of cellular processes on MDA-MB-231 cells after treatment with LRG1. The DEGs from MDA-MB-231 cells treated or not (control) with 100 ng/mL LRG1 were screened from transcriptome data, and Heatmap analysis was used to visualize the expression profiles of these DEGs during treatment with rhLRG1. Clusters of DEGs showing upregulation and downregulation are highlighted in red and blue. (**A**) Top 20 DEGs. The bar graph represents the cellular pathways and biological processes modulated after exposure to LRG1 (**B**) The results prominently highlight the enrichment of the PI3K-Akt and JAK-STAT signaling pathways. Likewise, pathways related to innate immunity are identified, including the signaling of RIG-I-like receptors, Toll-like receptors, and cytokine–receptor interactions. The values are represented by Fold Enrichment and the significance level (−log10 false discovery rate (FDR)).

The investigation into the therapeutic mechanism of rhLRG1 requires the comprehensive characterization of DEGs, specifically those associated with oncogenesis and tumor suppression. Therefore, we delineated the hub DEG profiles and ascertained their functional roles within the trajectory of breast carcinogenesis. Transcriptome data derived from MDA-MB-231 cells following exposure to rhLRG1 served as the foundational dataset for gene screening. Subsequent analysis employed Heatmap visualization to illustrate the expression landscape of the identified DEGs in response to the treatment. Specifically, the R Complex Heatmap tool was utilized to generate a global Heatmap, facilitating the clustering and visualization of gene expression profiles. This visualization stratifies genes into clusters based on their expression dynamics, highlighting upregulated genes (indicated in red and reddish) and downregulated genes (indicated in blue and bluish) (Figure 3). We found that 1920 genes maintained measurable expression across all designated time points following the rhLRG1 administration. Examination of the 20 representative DEGs demonstrated that their transcriptional levels varied in response to rhLRG1 (Figure 3A). Notably, all 20 representative DEGs showed uniform and marked upregulation after rhLRG1 treatment. Collectively, the Heatmap data provide compelling evidence of distinct expression changes among the 20 representative DEGs. These findings indicate that the substantial and generalized increase in gene expression observed post-treatment with rhLRG1 identifies critical and highly responsive hub DEGs within the molecular cascade of the MDA-MB-231 cellular response. Subsequently, the enriched cellular processes associated with these 20 main DEGs after modulation with LRG1 were determined (Figure 3B). Functional analysis highlighted the activation of key oncogenic signaling pathways, particularly the PI3K-Akt and JAK-STAT pathways. Likewise, a significant enrichment was observed in processes related to innate immunity and intercellular communication, including the signaling of RIG-I-like receptors, Toll-like receptors, and cytokine–receptor interaction. These findings suggest that rhLRG1 acts as a modulator of the inflammatory and survival response in this model of breast adenocarcinoma.

### 2.4. Functional Cellular Enrichment Analysis to Identify Cell Signaling Pathways Regulated by LRG1 in Breast Cancer Cells

**Figure 4:**
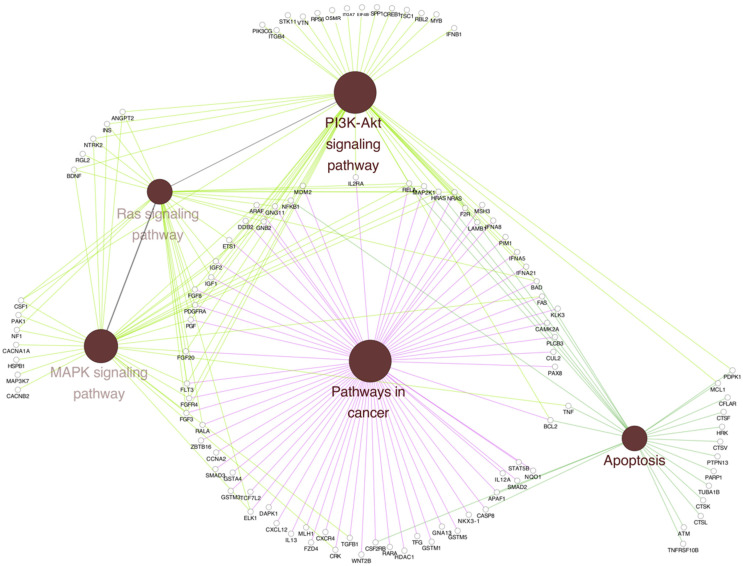
LRG1-induced enriched DEGs and cellular signaling pathways in MDA-MB-231 cells. Functional cellular enrichment analyses were conducted utilizing the Gene Ontology (GO) and the Kyoto Encyclopedia of Genes and Genomes (KEGG) platforms. Red points indicate the more relevant cellular signaling pathways activated by LRG1. PI3K-AKT, RAS, MAPK, and apoptosis signaling pathways are activated in MDA-MB-231 cells treated with rhLRG1. These cellular signaling pathways mainly regulate processes of cell cycle progression, proliferation, migration, and apoptosis. Up-expressed DEGs—*FGF8*, *ITGA7*, *PI3K*, and *HRAS*—are key in the regulation of these cellular processes.

**Figure 5:**
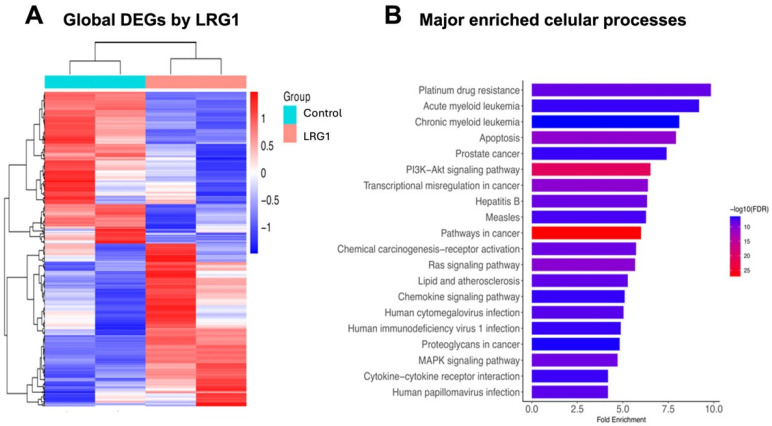
Major cancer-related cellular processes on MDA-MB-231 cells in response to LRG1. Enrichment analysis of cellular processes and cellular signaling pathways was performed using the Cluster Profiler and ggplot2 packages in R v4.0.2. The Kyoto Encyclopedia of Genes and Genomes (KEGG) database was used as the reference, with a false discovery rate (FDR) threshold of *p* < 0.05. Heatmaps were generated using the Complex Heatmap package in R v2.27.x, applying Pearson correlation analysis and complete clustering. (**A**) Global DEG expression in untreated (control) and hrLRG1-treated (100 ng/mL; LRG1) cells. Red and reddish colors indicate LRG1-activated signaling pathways. (**B**) Major enriched cellular processes highlighted the activation of PI3K/AKT, RAS, MAPK, and apoptosis signaling pathways following treatment with rhLRG1.

To explore plausible mechanisms involved in the effects of rhLRG1 treatment on MDA-MB-231 cells, comprehensive functional cellular enrichment analyses were conducted utilizing the Gene Ontology (GO) and the Kyoto Encyclopedia of Genes and Genomes (KEGG) platforms. These in silico analyses aimed to identify and characterize LRG1-modulated or -activated cell signaling pathways. The initial functional cellular enrichment analysis, focused on a subset of hub DEGs comprising known oncogenes and tumor-suppressor genes (Figure 4), revealed significant enrichment for the PI3K, RAS, and MAPK pathways. Additionally, upregulated key hub DEGs, including *IGF2*, *HRAS*, *FGF8*, and *ANGPT2*, were identified. A subsequent biologic-focused cellular enrichment analysis was performed to correlate the above-mentioned pathways with clinically relevant phenomena, such as drug resistance or the disruption of hub DEGs central to oncogenic processes (Figure 5). As a result, several breast cancer-specific hub DEGs with functional relevance to the modulation of key cancer progression hallmarks were identified (Figure 5A). Moreover, the analysis indicated enriched pathways including apoptosis, RAS signaling, chemical carcinogenesis-receptor activation, PI3K/AKT signaling, and general cancer-related signaling pathways (Figure 5B). Altogether, these findings support that rhLRG1 treatment induces significant transcriptional changes converging on signaling pathways able to regulate cellular proliferation, survival, and mechanisms of therapeutic resistance within the context of breast cancer.

**Figure 6:**
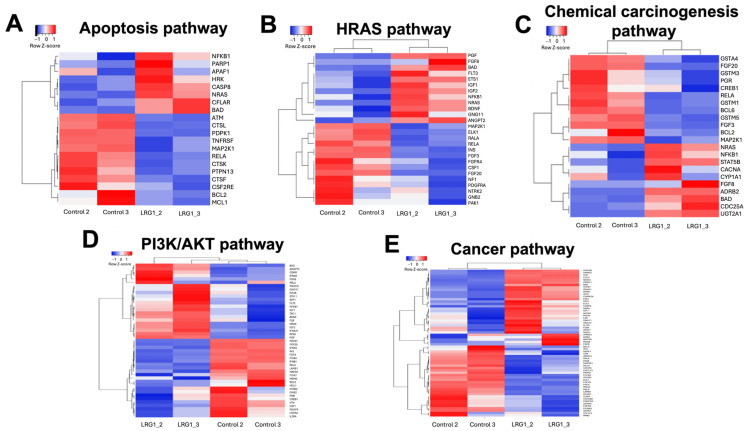
Analysis of hub DEGs in critical cell signaling pathways induced by LRG1 in MDA-MB-231 cells. The Heatmaps show the differential expression of hub DEGs related to different cellular processes in MDA-MB-231 cells treated with rhLRG1 compared to the untreated cells. Hub DEGs are grouped hierarchically according to similarities in their expression patterns, with colors indicating expression levels: red for up-expression and blue for down-expression. (**A**) Apoptosis, (**B**) HRAS, (**C**) chemical carcinogenesis-receptor activation pathway, (**D**) PI3K/AKT, and (**E**) general cancer-related signaling pathways.

Building upon the previous cellular enrichment findings (Figure 5), a targeted examination of hub DEGs (with a fold-change greater than 1) was performed to delineate LRG1-induced transcriptional modulations within the identified biological processes (Figure 6). The findings showed that hrLRG1 exerts a transcriptional DEG shift, suggesting an opposite regulation over pro-apoptotic activation genes associated with pro-apoptotic signaling and cell survival pathways in MDA-MB-231 (Figure 6A). Key examples of this activation include CASP8, which is a critical initiator caspase in the extrinsic (death receptor) apoptotic pathway; NRAS, which is implicated in various signaling pathways, with specific roles in promoting apoptosis depending on the cellular context; BAD, which is a pro-apoptotic member of the BCL-2 family that facilitates cytochrome C release; and MCL1, a critical anti-apoptotic protein that inhibits the function of pro-apoptotic BCL-2 family members. These data collectively support the notion that rhLRG1 can induce a pro-apoptotic effect in MDA-MB-231 cells. This transcriptional signature evokes an active shift in the cellular survival balance towards programmed cell death, representing a potential therapeutic mechanism of action for rhLRG1.

The analysis of hub DEGs within the PI3K signaling pathway revealed a complex and bifurcated pattern of gene regulation following treatment with rhLRG1, which may affect crucial aspects of cellular behavior and interaction with the tumor microenvironment. The overexpression of several hub DEGs driving critical cell signaling processes was observed. Specific overexpressed genes include *BAD*, *TSC1*, *ANGPT2*, and *PGF*; cell proliferation genes such as *NRAS* and *IGF2*; and protein synthesis genes such as *RPS6*. In contrast, the data also indicated the downregulation of other essential pathway components and genes related to the cellular microenvironment, potentially implying the inhibition of specific branches or functions. Moreover, the findings also showed downregulation of hub DEGs involved in cell adhesion and extracellular matrix interaction, including *ITGB4*, *ITGA7*, and *LAMB1*.

In rhLRG1-treated MDA-MB-231 cells, there was an over-expression of hub DEGs such as *BAD* and *STAT5B*, related to the regulation of apoptosis and cell proliferation, and *NRAS*, *IGF2*, and *NF-kB*, key activators of cell growth, proliferation signals, and survival through cascades such as MAPK and PI3K. Other hub DEGs found to be overregulated, such as *PGF* and *IFNA21*, are related to angiogenesis, indicating a promotion of the formation of new blood vessels essential for tumor progression. The overexpression of *SMAD2* and TGFb1 DEGs could regulate apoptosis and the modulation of the tumor microenvironment. In contrast, DEGs like *BCL2*, known for its role in inhibiting apoptosis, showed a reduction in expression, implying lower resistance to programmed cell death and the downregulation of *LAMB1*, *GSTM1*, and *GSTM5*. Hub DEGs responsible for interaction with the microenvironment and cellular detoxification were also observed, which could limit the ability of cells to resist chemotherapeutic agents. Furthermore, the decrease in hub DEGs related to the transduction of cell growth and proliferation signals, such as *FGF3*, *FGF20*, and *MAP2K*, may indicate attenuation of signals that favor tumor expansion.

### 2.5. Confirmation of Hub DEGs Profile in Breast Cancer Cells Treated with LRG1

**Figure 7:**
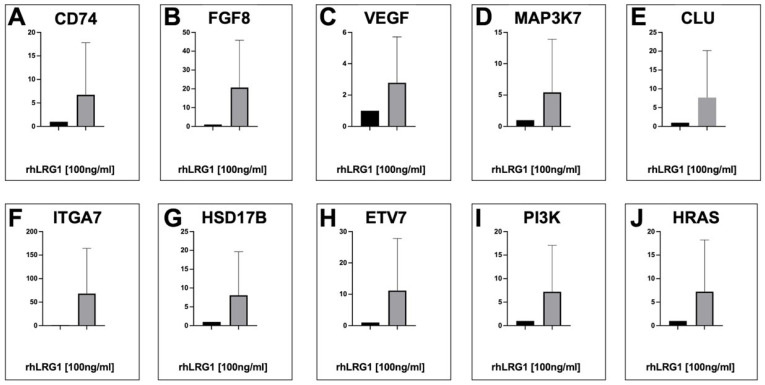
Analysis of DEGs by effect of LRG1 treatment on MDA-MB-231 cells. Quantitative real-time RT-PCR analysis of DEGs *CD74* (**A**), *FGF8* (**B**), *VEGF* (**C**), *MAP3K7* (**D**), *CLU* (**E**), *ITGA7* (**F**), *HSD17B* (**G**), *ETV7* (**H**), *PI3K* (**I**), and *HRAS* (**J**) on MDA-MB-231 cells in basal conditions as well as with 100 ng/mL rhLRG1 treatment protein. Total RNA and cDNA synthesis were obtained from 1 × 10^5^ cells per well in a six-well plate containing RPMI-1640 medium at 37 °C with 5% CO_2_. Relative expression by qRT-PCR analysis of DEGs was calculated using the 2^−ΔΔCt^ method and was analyzed by mRNA DEGs/mRNA beta-actin ratio relative expression units. Statistical analyses were performed with ΔCt values using an unpaired *t*-test with Welch correction and did not reveal a statistically significant difference between groups. The Ct values were normalized with untreated MDA-MB-231 cells and although an upward trend was observed for DEGs across biological replicates (n = 3), DEG expression was increased in treated cells compared to untreated cells.

To validate and confirm the data from rhLRG1-stimulated MDA-MB-231 cells observed in the transcriptomic analysis using microarrays, we analyzed the gene expression of specific DEGs by real-time qRT-PCR. We identified an increase in gene transcripts after 24 h of treatment with hrLRG1, ranging from 5- to 60-fold in comparison with untreated MDA-MB-231 cells. As shown in Figure 7, we selected the following specific DEGs: *CD74*, *FGF8*, *VEGF*, *MAP3K7*, *CLU*, *ITGA7*, *HSD17B*, *ETV7*, *PI3K*, and *HRAS* DEGs. Indeed, there was no statistically significant difference when comparing the groups (*CD74*, 6.7-fold and *p* = 0.9983; *FGF8*, 20.7-fold and *p* = 0.3502; *VEGF*, 2.8-fold and *p* = 0.9593; *MAP3K7*, 5.4-fold and *p* = 0.9326; *CLU*, 5.4-fold and *p* = 0.8901; *ITGA7*, 68.3-fold and *p* = 0.2635; *HSD17B*, 8-fold and *p* = 0.9136; *ETV7*, 11-fold and *p* = 0.8633; *PI3K*, 7.2-fold and *p* = 0.7672; and *HRAS*, *7*.2-fold and *p* = 0.8687). The housekeeping gene expression level of beta-actin did not show any changes under these same conditions. Although we did not observe statistically significant differences, our data indicate that LRG1 is protein-specific and has the ability to induce selective and specific hub DEGs involved in PI3K, RAS, MAPK, and apoptosis signaling pathways, as well as in pathways in cancer, in human breast cancer cells, which can explain, in part, the progression of the breast carcinogenesis process.

## 3. Discussion

Breast cancer remains a major global health concern. Significant challenges persist in identifying and implementing treatments capable of achieving complete disease remission and preventing cancer recurrence [35,36]. LRG1 protein has been extensively documented in the literature for its role in modulating various oncogenic cellular processes, including proliferation, migration, invasion, and metastasis. In the context of breast cancer, LRG1 functions as a significant diagnostic and prognostic biomarker, with elevated protein levels correlating with both early and advanced stages of malignancy, as well as reduced disease-free survival and overall survival [37]. The current investigation corroborates and extends these prior observations by demonstrating that exogenous treatment with rhLRG1 directly increases the viability and enhances the migratory capacity of the MDA-MB-231 breast cancer cell line. Furthermore, this study successfully identified key hub DEG profiles that elucidate the molecular mechanisms underlying this biological impact. These transcriptional analyses point to the modulation of cellular signaling pathways centrally involved in the development and progression of breast tumors. In particular, the observed effects on cell viability and migration, coupled with the transcriptional changes in pathways related to apoptosis, proliferation, and microenvironment interaction, position LRG1 as an interesting factor in driving the aggressive phenotype of breast cancer cells.

LRG1 has been described to influence cellular regulatory mechanisms through the SRC/STAT3/VEGFA, TGF-β1, and RUNX1 signaling pathways [25,31,38]. We demonstrated herein that LRG1 enhances both cell viability and migration in MDA-MB-231 cells (Figure 1 and Figure 2). Indeed, there is a positive correlation between LRG1 expression and breast cancer prognosis [22]. An elevated expression of LRG1 was observed in breast cancer samples, and LRG1 expression was associated with the number of lymphatic metastases [22]. In this regard, our present findings provide significance to the increased expression of LRG1 in immortal and tumorigenic cells, which closely resemble malignant breast lesions.

To understand the LRG1 regulatory genetic network, we analyzed the transcriptomic data obtained from the microarray assays in MDA-MB-231 cells (Figure 3) and performed a cellular enrichment analysis to identify target cell signaling pathways (Figure 4). The results identified hub DEGs involved in the activation of signaling pathways relevant to cancer progression, such as PI3K, MAPK, and RAS. Whereas these findings agree with studies from other research groups [39,40], our findings are novelty in reporting upregulated hub DEGs involved in cell proliferation (*NRAS*, *STAT5B*, *IGF2*), angiogenesis (*PGF*, *ANGPT2*), and survival (*CASP8*, *BAD*), and downregulated ones regulating functions such as apoptotic resistance (*BCL2*, *MCL1*) and cell adhesion (*LAMB1*, *ITGB4*).

Biologically focused cellular enrichment analysis revealed the activation of key processes in cancer progression, such as apoptosis, PI3K signaling, transcriptional dysregulation in cancer, RAS signaling, MAPK signaling, and general cancer-related signaling pathways. Consequently, this finding reinforces the role of LRG1 treatment in modulating these key molecular pathways (Figure 5).

We identified upregulated and downregulated key hub DEGs regulating biological processes (Figure 6). For the former, *ANGPT2*, *BAD*, *CASP8*, *CD74*, *CLU*, *ETV7*, *FGF8*, *HRAS*, *HSD17B*, *IGF2*, *MAP3k7*, *NRAS*, *STAT5b*, *TSC1*, *PGF*, *RPS6*, *PI3K*, *PDPK1*, *ITGB4*, *ITGA7*, *LAMB1*, *IFNA21*, *SMAD2*, *TGF-β1*, and *VEGF* were found, which are of interest due to their involvement in the regulation of pro-apoptotic signaling and cell survival.

The angiopoietin-2 (ANGPT2) expression levels are proportional to the cancer stage for both small and non-small cell lung cancers. It has also been implicated in hepatocellular and endometrial carcinoma-induced angiogenesis [41]. The BCL2-associated agonist of cell death (BAD) protein is a pro-apoptotic member of the Bcl-2 gene family, which is involved in initiating apoptosis [42]. BAD is a member of the BH3-only family, a subfamily of the Bcl-2 family. It lacks a C-terminal transmembrane domain for outer mitochondrial membrane and nuclear envelope targeting [42]. After activation, BAD is able to form a heterodimer with anti-apoptotic proteins and prevent them from stopping apoptosis. *CASP8* gene encodes a member of the cysteine–aspartic acid protease (caspase) family. Sequential activation of caspases plays a central role in the execution phase of cell apoptosis [43]. Recently, it has been reported that CASP8 may potentially serve as a biomarker for high-risk prostate cancer and possibly renal cancer [44].

CD74 (HLA gamma chain or HLA-DR invariant chain) is a polypeptide which plays a critical role in antigen presentation for generation of CD4+ T cell responses [45]. CD74 is a cell surface receptor for the cytokine macrophage migration inhibitory factor (MIF). Recently, it has been reported that MIF was highly expressed in cancer-associated fibroblasts and cancer cells from pancreatic ductal adenocarcinoma [46]. CLU is an extracellular molecular chaperone protein which binds to misfolded proteins in body fluids to neutralize their toxicity and mediate their cellular uptake by receptor-mediated endocytosis. There is evidence identifying CLU as an upstream regulator of pro-metastatic adhesion–cytoskeleton signaling in breast cancer cells. [47]. E26 transformation-specific variant transcription factor 7 (ETV7) is a member of E26 transformation-specific (ETS) transcription factor family. ETS networks interact with co-regulatory partners to elicit gene-specific responses and drive distinct biological processes. ETV7 is overregulated in several malignancies. In breast cancer, ETV7 suppresses inflammation by downregulating TNFRSF1A/NF-kB axis [48].

The *HRAS* and *NRAS* proto-oncogenes are members of the *RAS* gene family. These *RAS* genes have GTP/GDP binding and GTPase activity, and their normal function may be as G-like regulatory proteins involved in the normal control of cell growth [49]. *HRAS* mutations are associated with increased PD-1/PD-L1 and TCF1 expression, inducing a potential sensitivity to immunotherapy in head and neck squamous-cell carcinoma [50]. Fibroblast growth factor 8 (FGF8) is a protein that belongs to the fibroblast growth factor (FGF) family. FGF proteins are multifunctional signaling molecules with broad mitogenic and cell survival activity, playing critical roles in embryonic development, cell proliferation, morphogenesis, tissue repair, and tumor progression [51]. It has been reported that FGF8, and associated hub DEGs, help in the progression of ovarian cancer, and their overexpression may lead to higher immune infiltration, poor prognosis, and poor survival [52]. Insulin-like growth factor 2 (IGF2) is one of three protein hormones that share structural similarity to insulin. IGF2 is sometimes produced in excess in islet cell tumors and non-islet hypoglycemic cell tumors, causing hypoglycemia. Doege–Potter syndrome is a paraneoplastic syndrome in which hypoglycemia is associated with the presence of one or more non-islet fibrous tumors in the pleural cavity [53].

17β-Hydroxysteroid dehydrogenase (HSD17B, 17β-HSD or 17-KSR) is an alcohol oxidoreductase which catalyzes the reduction of 17-ketosteroids and the dehydrogenation of 17β-hydroxysteroids in steroidogenesis and steroid metabolism. Previous evidence indicates that transcription factor GATA1 activates HSD17B6 to inhibit cisplatin resistance in lung adenocarcinoma through DNA damage. Thus, the GATA1/HSD17B6 axis may be a potential therapeutic target for chemotherapy resistance in lung adenocarcinoma patients [54]. Signal transducer and activator of transcription type 5B (STAT5B) is a protein that belongs to STAT family of transcription factors [55]. STAT family members are phosphorylated by receptor-associated kinases and then form homo- or heterodimers that translocate to the cell nucleus where they act as transcription activators [55,56]. Tuberous sclerosis 1 (TSC1 or hamartin) is a protein that functions as a co-chaperone inhibiting the ATPase activity of the chaperone Hsp90 and functions as a facilitator of Hsp90 in chaperoning the kinase and non-kinase clients, preventing their ubiquitination and degradation in the proteasome [57]. TSC1, TSC2 and TBC1D7 form the TSC complex, which regulates mTORC1 signaling by functioning as a GTPase-activating protein (GAP) for the small GTPase Rheb, an essential activator of mTORC1. The TSC complex has been implicated as a tumor suppressor that has potential downstream impact of this regulation on normal cellular function and in human cancers [58].

The placental growth factor gene (*PGF*) is a member of the vascular endothelial growth factor (VEGF) family. PGF is expressed only in human umbilical vein endothelial cells, and the placenta. PGF is ultimately associated with angiogenesis during carcinogenesis processes [59]. Ribosomal protein S6 (RPS6, or eS6) is a component of the 40S ribosomal subunit and is therefore involved in translation [60]. Studies in mice have shown that phosphorylation of RPS6 regulates cell size, cell proliferation, and glucose homeostasis [60]. Phosphorylated RPS6 (p-RPS6) has been reported as the most differentially expressed protein between metastatic and non-metastatic colorectal cancer tissues, which was found to be positively correlated with EMT proteins and poor prognosis in colorectal cancer [61]. The 3-phosphoinositide-dependent protein kinase-1 (PDPK1) is a kinase for activation of AKT/PKB and many other AGC kinases, including PKC, S6K, and SGK. An important role for PDPK1 is in the signaling pathways activated by several growth factors and hormones, including insulin signaling; thus it is implicated in cellular processes including survival, metabolism and tumorigenesis. Previous reports have reported that responsiveness to PI3K pathway inhibition is associated with decreased expression of pigmentation genes and increased expression of cytokines and inflammatory genes, alluding to a method to stratify patients with melanoma for PI3K-based therapies [61].

Integrin beta 4 (*ITGB4* or *CD104*) is a gene that encodes the integrin beta 4 subunit. Integrins are heterodimers composed of alpha and beta subunits that are noncovalently associated transmembrane glycoprotein receptors. In hepatocarcinoma, it has been reported that PD-L1 directly interacts with ITGB4 to activate the FAK/AKT/mTOR signaling pathway, independent of its immune-regulatory functions. This interaction critically mediates sorafenib resistance [62]. Alpha-7 integrin or ITGA7 is an integrin critical for modulating cell–matrix interactions. In gliomas, key integrin-mediated genes have been identified that significantly contribute to poor prognosis through a combined approach of machine learning and protein–protein interaction. Through this network analysis it was demonstrated that ITGA7 can serve as a biomarker for gliomas [63].

Laminin subunit beta-1 (LAMB1) belongs to the laminins family which are extracellular matrix glycoproteins and are the major non-collagenous constituent of basement membranes. It has been reported that LAMB1 overexpression enhances cell viability, proliferation, and invasion and promotes glioma cell growth through regulation of the NF-κB/HK2 axis [64]. IFNA21 belongs to type I interferon-alpha (IFN-alpha) [65]. A comprehensive analysis to investigate the expression patterns and functional enrichment of interferon-related genes (IRGs) in glioma has been reported [61]. Notably, IFNA21 was markedly downregulated in glioma tissues compared to normal tissues, and higher expression level was associated with improved overall survival and disease-specific survival [66].

Mothers against decapentaplegic homolog 2 (SMAD2) mediates the TGF-β1 signaling pathway, regulating multiple cellular processes, such as cell proliferation, apoptosis, and differentiation [67]. TGF-β1 is a polypeptide member of the TGF-β cytokine superfamily. It is a secreted protein that performs many cellular functions, including the control of cell growth, cell proliferation, cell differentiation, and apoptosis. Modulatory influences on prognosis, tumor microenvironment, and therapeutic efficacy of TGF-β1 have been studied in colorectal cancer [68]. The evidence illustrates transcriptional and genetic alterations of TGF-β1 relevant genes, which are closely linked with carcinogenic pathways. Moreover, findings have allowed the construction of a novel TGF-β1 scoring model that could predict prognosis, liver metastasis tendency, and tumor microenvironment characteristics for colorectal cancer patients. Otherwise, TGF-β2 triggers the downstream SMAD2/3 signaling, whose inhibition by simvastatin (an HMG-CoA inhibitor) could enhance the cytotoxicity of NK cells against endometrial cancer cells [67].

Glutathione S-transferase Mu-1 and -5 (GSTM1 and GSTM5) are human glutathione S-transferases belonging to the mu class of enzymes that function in the detoxification of electrophilic compounds, including carcinogens, therapeutic drugs, environmental toxins, and products of oxidative stress, by conjugation with glutathione [69,70]. Null mutations of this mu class gene have been linked with an increase in several cancers, likely due to an increased susceptibility to environmental toxins and carcinogens [69]. On the other hand, mRNA expression levels of GSTM1-5 are substantially higher in estrogen-receptor-positive and progesterone-receptor-positive patients compared to receptor-negative patients with breast cancer [70]. Vascular endothelial growth factor (VEGF) is a signal protein produced by many cells that stimulates the formation of blood vessels. VEGF is a sub-family of growth factors from platelet-derived growth factor family of cystine-knot growth factors. They are important signaling proteins involved in angiogenesis processes. Previously the interaction between curcumin and IL-1β for VEGF secretion in breast cancer cell lines representing major breast cancer subtypes have been reported [71].

We also identified DEGs showing low expression, like *BCL-2* and *MCL1* able to inhibit apoptosis. BCL2 blocks programmed cell death by apoptosis, while other BCL-2 family members can either inhibit or induce it [72]. Gene-expression profiling revealed that *CDK11* knockdown significantly downregulated the anti-apoptotic factor BCL-2 [73]. Furthermore, studies show that overexpression of BCL-2 partially reversed the autophagy and the inhibition of proliferation and migration induced by *CDK11* knockdown [73]. Induced myeloid leukemia cell differentiation (MCL1) is a protein that belongs to Bcl-2 family. Alternative splicing occurs at this locus and two transcript variants encoding distinct isoforms have been identified. The longer gene product (isoform 1) enhances cell survival by inhibiting apoptosis, while the alternatively spliced shorter gene product (isoform 2) promotes apoptosis and is death-inducing [74]. Recently, it has been reported that MCL1 has a novel regulatory function in determining breast cancer-associated fibroblast subpopulation differentiation, and its role in modulating their pro-angiogenic properties, in response to treatment, has been highlighted [74].

Interestingly, within the RAS signaling pathway, we observed overexpression of key hub DEGs such as *BDNF*, *ANGPTL4*, *RELA*, *FGF3*, *FGF20*, *PDGFR*, *NF-κB*, *MAPK* in MDA-MB-231 cells treated with LRG1. Brain-derived neurotrophic factor (BDNF or abrineurin) is a member of the neurotrophin family of growth factors, which are related to the canonical nerve growth factor (NGF), a family which also includes NT-3 and NT-4/NT-5. Recently it has been reported that a dysregulated cytokine signaling and altered BDNF expression in cancer-related cognitive impairment among young adult cancer patients during and post-chemotherapy [75]. Angiopoietin-like 4 is a protein that is encoded by the *ANGPTL4* gene. Alternatively spliced transcript variants encode different isoforms [76]. This gene is induced under hypoxic conditions in various cell types and is the target of peroxisome proliferator-activated receptors. The encoded protein is a serum hormone directly involved in regulating lipid metabolism. ANGPTL4 plays an important role in numerous cancers and is implicated in the metastatic process by modulating vascular permeability, cancer cell motility and invasiveness [76]. RELA, or p65, is a REL-associated protein involved in NF-κB heterodimer formation, nuclear translocation, and activation [77]. Phosphorylation and acetylation of RELA are crucial post-translational modifications required for NF-κB activation. NF-κB/RELA axis activation has been found to be correlated with cancer development, indicating the potential of RELA as a cancer biomarker. Specific modification patterns of RELA have also been observed in many cancer types [78].

FGF3 and FGF20 are members of fibroblast growth factor family. FGF3 binds to fibroblast growth factor receptor 3 (FGFR3) to serve as a negative regulator of bone growth during ossification. Amplification of the FGF3 gene has been found in human tumors, which may be important for neoplastic transformation and tumor progression [79]. FGF3 and FGF20 are involved in embryonic development, cell growth, morphogenesis, tissue repair, tumor growth, and invasion in different pathological stages of head and neck squamous cell carcinoma [80,81]. Additionally, it has been demonstrated that FGF20 modulates glucocorticoid-induced dysfunction of macrophages during glioma development [82]. Platelet-derived growth factor receptors (PDGFRs) are cell surface tyrosine kinase receptors for members of the platelet-derived growth factor (PDGF) family. PDGF subunits -A and -B are important factors regulating cell proliferation, cellular differentiation, cell growth, development, and many diseases, including cancer [83]. It has been described how the screening of a panel of kinase inhibitors (KIs) identified the PDGFR-alpha/beta inhibitor CP-673451 as a potential differentiation agent in glioblastoma through DUSP1/p38 MAPK signaling [84].

Nuclear factor kappa-light-chain-enhancer of activated B cells (NF-κB) is a family of transcription factor protein complexes that regulate transcription of DNA, cytokine production, and cell survival, and is involved in several cellular processes, including metabolism, chemotaxis, and immune response. Incorrect regulation of NF-κB has been linked to cellular transformation, viral pathogenesis and cancer development [85]. Many different types of human tumors have dysregulated NF-κB, which is the constitutively active form. Active NF-κB triggers the expression of genes that keep the cell proliferating and protect the cell from conditions that would otherwise cause it to die via apoptosis. This is evident both in metastasis and in the inefficient eradication of the tumor by the immune system [85]. Mitogen-activated protein kinase kinase (MAP2K, MEK or MAPKK) is a dual-specificity kinase enzyme which phosphorylates mitogen-activated protein kinase (MAPK). A MAPK or MAP kinase is a type of serine/threonine-specific protein kinase involved in directing cellular responses to a diverse array of stimuli, such as mitogens, osmotic stress, heat shock, and proinflammatory cytokines. They regulate cell functions including proliferation, gene expression, differentiation, mitosis, cell survival, and apoptosis [86]. It has been identified that MEK inhibitors suppress radiation-induced activation of RAS-MAPK signaling and selectively downregulate components of the homologous recombination DNA repair pathway [87].

Finally, we validated and confirmed the data observed in the transcriptomic analysis. Interestingly, we identified an increase in the gene transcript level of the *CD74*, *FGF8*, *VEGF*, *MAP3K7 CLU*, *ITGA7*, *HSD17B*, *ETV7*, *PI3K*, and *HRAS* DEGs when MDA-MB-231 cells were treated with rhLRG1 protein (Figure 7). These data demonstrate that LRG1 is a protein that has the ability to induce selective and specific hub DEGs involved in cellular signaling pathways in human breast cancer cells, which can explain, in part, the progression of breast carcinogenesis processes.

A limitation of this study is that pathway activation was inferred based on mRNA expression levels. While we observed changes in the expression of genes associated with PI3K/AKT and MAPK signaling pathways, it is well established that these pathways are primarily regulated at the post-translational level, particularly through protein phosphorylation and activation states. Therefore, mRNA expression alone is not sufficient to confirm pathway activation. Further studies, including protein-level analyses such as Western blotting, are required to validate the activation status of these signaling pathways.

Regarding hub DEGs involved in the PI3K signaling pathway, upregulation of DEGs involved in cell signaling processes is observed, such as the regulation of apoptosis (*BAD*, *TSC1*), angiogenesis (*ANGPT2*, *PGF*), cell proliferation (*NRAS*, *IGF2*) and protein synthesis (*RPS6*). The activation of these DEGs demonstrates that LRG1 treatment favors processes that promote cell growth and adaptation in tumor environments. While the downregulation of *PDPK1* and *MAP2K1*, essential components in signal transduction through *PI3K*, could induce inhibition of certain branches of this pathway, the downregulation of genes related to cell adhesion and interaction with the microenvironment, such as *ITGB4*, *ITGA7*, and *LAMB1*, could represent an alteration in the ability of cells to interact with their tumor microenvironment.

In the hub DEGs from the cancer signaling pathway, the Heatmap shows that among the upregulated DEGs, *NRAS* and *IGF2* stand out as central regulators involved in cell proliferation and survival through cascades such as MAPK and PI3K. Also, the *STAT5B* and *NF-κB* DEGs showed upregulation, contributing to inflammation, proliferation, and resistance to apoptosis. In addition, the upregulation of *PGF*, *IFNA21* and *VEGF* was identified, DEGs related to angiogenesis, indicating a promotion of the formation of new blood vessels essential for tumor progression. Additionally, the *SMAD2* and *TGF-β1* DEGs were upregulated, indicating a role in the regulation of apoptosis and the modulation of the tumor microenvironment. In contrast, *BCL2*, known for its role in inhibiting apoptosis, showed a reduction in its expression, indicating a lower resistance to programmed cell death. The downregulation of *LAMB1*, *GSTM1*, and *GSTM5* DEGs responsible for interaction with the microenvironment and cellular detoxification, was also observed, which could limit the ability of cells to resist chemotherapeutic agents.

In the analysis of hub DEGs in the RAS signaling pathway, we observed upregulation of DEGs involved in the regulation of apoptosis, cell proliferation and survival, and angiogenesis, which could indicate that LRG1 modulates the RAS signaling pathway in a complex way, enhancing DEGs associated with pro-apoptotic and angiogenic functions, while attenuating the expression of DEGs involved in proliferative and inflammatory signals. RAS is a small GTPase that cycles between GTP-bound (active) and GDP-bound (inactive) states and binds to downstream effector proteins to activate RAF/MEK/ERK and PI3K/AKT–mTOR proliferative pathways. The three RAS isoforms include HRAS, NRAS, and KRAS; collectively, they are one of the most mutated cancer targets, with over 3 million cancer patients diagnosed with RAS mutations each year. The reduction in their expression levels evokes an inhibitory effect of the treatment on certain branches of the RAS signaling pathway that may favor breast tumor progression. Thus, a long-standing goal in the field has been to develop RAS therapies. This thin balance could have important implications for the dynamics of the tumor microenvironment.

In the hub DEGs of the *PI3K* and *MAPK* signaling pathways, a clear upregulation of DEGs involved in key cell signaling processes is observed, such as the regulation of apoptosis, angiogenesis, cell proliferation, and protein synthesis. The PI3K/Akt/mTOR pathway appears to be a prime target. It involves a signaling cascade beginning with PI3K activation followed by activating phosphorylation of Akt and then mTOR complex, which activates oncogenic processes by enhancing protein synthesis, inhibiting apoptosis, dysregulating autophagy and promoting DNA repair that supports tumor cell survival [62]. MAPK pathways are central signaling elements that transmit signals through sequential activation of three to five layers of protein kinases known as MAPK kinase kinase kinase (MAP4K), MAPK kinase kinase (MAP3K), MAPK kinase (MAPKK), MAPK and MAPK-activated protein kinase (MAPKAPK) [88]. Although we consider that mRNA expression alone is insufficient and represents a limitation to drawing conclusions about the activation of the PI3K/AKT and MAPK pathways, which are regulated post-translationally by phosphorylation status, activation of these DEGs strengthens the notion that LRG1 treatment favors processes that promote cell growth and adaptation in tumor environments. We consider that more studies are necessary to identify phosphorylation protein status of PI3K/AKT and MAPK.

On the other hand, the overexpression of PDPK1 and MAP2K1, essential components in the transduction of signals through PI3K, could mean that there is an inhibition of certain branches of this signaling pathway, while the reduction in *BCL2* and *MCL1*, known for their role in inhibiting apoptosis, indicates a greater sensitivity of the treated cells to pro-apoptotic signals. This regulation of the hub DEGs profile provides evidence that LRG1 treatment modulates the PI3K signaling pathway in a complex way, promoting signals associated with proliferation, survival, and angiogenesis while attenuating genes related to apoptosis resistance and cell adhesion.

These biological events appear to be paradoxical findings. However, it has been previously described that at the extracellular level, LRG1 binds to TGF-β1, activating the canonical TGF-β1 pathway; this signaling pathway is pro-apoptotic, but studies on SW480 and HCT116 cell lines of colorectal cancer also reported, after the overexpression of LRG1, an increase in the expression of RUNX1, cyclins D1, B and E, and BCL2, as well as a decrease in the levels of Bax and cleaved caspase 3. Conversely, inhibition of RUNX1 in these same cell lines reversed the expression of these proteins, increasing Bax and cleaved caspase 3, while decreasing cell cycle proteins and BCL2. These results confirm that RUNX1 regulates LRG1-mediated apoptosis [88].

In addition, in another study, in the MCF-7 cell line of breast adenocarcinoma, it was identified that intracellular LRG1 binds to Cytochrome C; this binding inhibits the interaction between Cytochrome C and Apaf-1, compromising the formation of the apoptosome and interfering with the activation of caspase 9 and the common pathway of apoptosis [89]. Thus, our data and other findings reported by different research groups support the notion that LRG1 can activate several signaling pathways, depending on the biological context, influenced by factors such as Cytochrome C or TGF-β1 binding to LRG1.

Taken together, our data and other findings reported by different research groups support the notion that LRG1 can activate several signaling pathways, depending on the biological context. Thus, these findings propose a multifaceted impact of treatment on tumor dynamics. Our data are in agreement with other research groups. In this sense, previous studies on different tumor types, like pancreatic [24], gastric [25], colorectal [26,90], and breast cancer [23], have identified that LRG1 regulates critical hub DEGs, as well as promoting tumor growth and progression through the hub DEGs involved in the apoptosis pathway, the RAS signaling pathway, the chemical carcinogenesis-receptor activation pathway, the PI3K/AKT signaling pathway, and general cancer-related signaling pathways.

## 4. Materials and Methods

### 4.1. Cell Line and Culture Conditions

The human breast cancer cell line MDA-MB-231 was sourced from the American Type Culture Collection (ATCC). This line was maintained under established culture conditions to ensure viability and standardized cellular behavior. Cells were routinely cultured in RPMI-1640 medium (Invitrogen, Carlsbad, CA, USA), which was supplemented with 10% Fetal Bovine Serum (FBS), 50 μg/mL penicillin/streptomycin, 2 mM L-glutamine, and 250 ng/mL fungizone. The culture environment was strictly controlled at 37 °C within a humidified incubator supplied with 5% CO_2_. The cultured MDA-MB-231 cells served as the biological substrate for a comprehensive set of functional and molecular analyses, such as cell viability, cell migration, qRT-PCR, and transcriptomic assays. The resulting data were subsequently subjected to rigorous bioinformatic analysis, including cellular enrichment processes and network construction and analysis.

### 4.2. Crystal Violet Staining

Crystal violet staining is commonly used to indirectly evaluate cell proliferation and viability in mammalian cell cultures. As cells die, they detach from the surface, reducing the overall staining intensity, which serves as an indicator of cell viability. Crystal violet staining dye works by binding to the cells’ negatively charged molecules, such as nucleic acids and proteins. We prepared a 0.1% (*w*/*v*) crystal violet in phosphate-buffered saline (PBS). After preparing the cells, we applied the solution of crystal violet to the cells. We allowed crystal violet to stain for 15–30 min at room temperature. We fixed the cells by gently washing with PBS, followed by treatment with 1% ethanol. After washing and drying the stained cells, solubilized the bound dye with 1% ethanol. We measured the absorbance of the solubilized dye at 590 nm. Documentation was performed via microphotography using a Vista Vision inverted microscope (VWR, Radnor, PE, USA) with a 100× objective.

### 4.3. Cell Viability Assays

Cellular viability was measured using a [3-(4,5-dimethylthiazol-2-yl)-5-(3-carboxymethoxyphenyl)-2-(4-sulfophenyl)-2H-tetrazolium] inner salt MTT assay (Promega, Madison, WI, USA), which is a colorimetric method for determining the number of viable cells in a proliferation or cytotoxicity assay. Briefly, a total of 5 × 10^4^ MDA-MB-231 cells per well were seeded in a 24-well plate. MDA-MB-231 cells were treated with 10 ng/mL or 100 ng/mL of rhLRG1 protein (Sino Biological, cat: 13371-H08H, Houston, TX, USA), incubated at 37 °C with 5% CO_2_. After 24 h of plating, 20 mg/mL MTT reagent was added to each well containing the untreated and treated cells, and cultures were incubated for 2 h. The MTT tetrazolium-compound salt reagent was bio-reduced by living cells into a colored formazan product that was insoluble in tissue culture medium. The supernatant was then aspirated, and 250 µL of DMSO was added to each well to dissolve the formazan crystals. After incubation, the absorbance values were measured at 540 nm using a microplate reader (ThermoFisher Scientific. Waltham, MA, USA). Cellular viability rate was calculated as the percentage of MTT adsorption as follows: survival percentage (%) = (mean experimental absorbance/mean control absorbance) × 100. The data were analyzed with the program GraphPad Prism 9.0 (GraphPad, San Diego, CA, USA). All assays were repeated at least three times independently.

### 4.4. Cellular Migration Assays

The MDA-MB-231 breast cancer cells were seeded into 6-well plates at a density of 3.5 × 10^4^ cells per well. Cells were cultured in RPMI-1640 medium supplemented with 10% FBS and maintained until a confluent monolayer of approximately 90% was obtained. Once confluence was reached, a uniform linear wound (stria) was physically introduced into the cellular monolayer by gently scraping the surface with the tip of a 10 μL micropipette. To remove cellular debris and any non-adherent or damaged cells generated during the scraping process, the cultures were rinsed twice with PBS. Following the creation of the wound, the cells were incubated at 37 °C in fresh RPMI-1640 medium containing 10% FBS. Experimental conditions were established as follows: untreated cells group received only the RPMI-1640 medium; treated cells were exposed to recombinant human LRG1 (rhLRG1) protein at concentrations of 10 ng/mL or 100 ng/mL. The process of cellular migration towards the center of the induced wound was systematically documented following 0, 6, 12, and 24 h after the initial scrape. The documentation was performed via microphotography using a Vista Vision (VWR, Radnor, PE, USA) inverted microscope with a 100× objective. The entire cellular migration assay was executed, and all assays were repeated at least three times independently.

### 4.5. Analysis of Hub Differentially Expressed Gene (DEG) Profile

MDA-MB-231 cells were seeded at a density of 7 × 10^4^ cells in 100 mm culture plates. After a 24 h adherence period, experimental groups were established: untreated cells received standard RPMI-1640 medium, and rhLRG1-treated cells were stimulated with 100 ng/mL. Both groups were incubated for an additional 24 h at 37 °C under 5% CO_2_ conditions. Total RNA was isolated using the guanidine/phenol-chloroform isothiocyanate reagent (Trizol) (Roche, Indianapolis, IN, USA). The integrity and purity of the isolated RNA samples were rigorously validated before expression analysis. RNA concentration was measured, and purity was assessed using the Thermo Scientific Micro-UV/Vis NanoDrop One spectrophotometer (CTR Scientific, Monterrey, Mexico). RNA integrity was confirmed by analysis via 1% agarose gel electrophoresis.

Once the quality-controlled RNA samples were obtained (in three independent biological replicates), gene expression analysis was performed using microarray technology in collaboration with the Microarray Unit of the Institute of Cellular Physiology at the National Autonomous University of Mexico (UNAM). The samples were hybridized to the HCa_05_08 chip (MWG Biotech, High Point, NC, USA), which contains probes for 1920 genes specifically related to human cancer, sourced from the SAGE library. Fluorescent labeling was performed using distinct fluorophores for differentiation. Untreated cells were labeled with ALEXA 555 and rhLRG1-treated samples were labeled with ALEXA 647, in two independent experiments. The raw hybridization intensity data was found using the GenArise R package version 1.86.0 software using default parameters established on developer manual (https://bioconductor.org/packages/genArise (accessed on 6 February 2026).

### 4.6. Bioinformatic and Computational Analysis

Gene expression intensity data were utilized for bioinformatic analysis. Data processing and statistical computations were conducted within the RStudio integrated development environment (IDE v2025.09) utilizing the R programming language (version v4.2.1). For data organization and initial manipulation, the Microsoft Excel spreadsheet software was employed. The construction, visualization, and specialized analysis of the molecular interaction networks, specifically networks, were performed using the Cytoscape software (version v3.10.3) [91].

### 4.7. Microarray Data Analysis

Fluorescence intensities microarray data were normalized and analyzed using the Z-score method algorithm with Microsoft Excel software [92]. The genes with a Z-ratio greater than −/+ 1.5 were considered statistically significant (DEGs). The datasets supporting the conclusions of this article are available in the [n9.cL] repository [unique persistent identifier and hyperlink to datasets: https://n9.cL/t9tg4t (accessed on 6 February 2026)]. Furthermore, we used the microarray platform HCa-05-08 chip library SAGE (MWG Biotech AG, Ebersberg, Germany).

### 4.8. Cellular Enrichment Processes and Pathway Analysis

The DEG results were integrated in a functional enrichment analysis of cellular processes and molecular pathways systematically conducted using the R statistical environment, specifically leveraging the ClusterProfiler v4.12.x and ggplot2 packages v4.0.2 according to their developer guidelines. The functional annotation was performed using two primary databases: Gene Ontology (GO) was used to detect DEGs based on their involvement in biological processes, cellular components, and molecular function [93]. Kyoto Encyclopedia of Genes and Genomes (KEGG) was utilized as reference database for identifying and mapping the hub DEGs to annotated signaling and metabolic pathways [94]. For statistical stringency, a false discovery rate threshold of *p* < 0.05 was applied to all enrichment results, ensuring a controlled rate of false positives. For advanced visualization and pattern identification, Heatmaps clustering was generated using the Complex Heatmap package in R v2.27.x, applying Pearson correlation and complete distribution. All analyses adhered to the default parameters recommended by the package developers.

### 4.9. Construction and Analysis of Hub DEG Network

The construction of the hub DEG network, and its subsequent cellular enrichment analysis, were performed using the Cytoscape software (version 3.10.3) [91]. For specialized functional annotation and visualization within the network context, the ClueGo and CluePedia plugins were utilized. The input data matrix for the network analysis was strictly limited to the hub DEGs identified from the microarray analysis. Each hub DEG was accompanied by its corresponding Z-score value to quantify the magnitude and direction of expression change. The network was constructed and analyzed against the KEGG database [87], which served as the primary reference for pathway mapping. A stringent statistical threshold of false discovery rate <0.05 was applied to all analyses to define significant interactions and enrichment terms.

### 4.10. Quantitative Real-Time RT-PCR Analysis of DEGs

Total RNA isolation from MDA-MB-231 cells was carried out with guanidine/phenol-chloroform (Trizol) isothiocyanate reagent (Roche, Indianapolis, IN, USA). The Script cDNA kit (Jena Biosciense, Jena, Germany) and Maxima Sybr Green Rox kit (Thermo Scientific, Waltham, MA, USA) were used for qRT-PCR assays. The qRT-PCR reaction started with an initial incubation step of at 95 °C for 2 min to activate the DNA polymerase. The one-step PCR cycling protocol, which has a denaturation step at 95 °C for 5 s and a combined annealing/extension step at 60 °C for 10 s, was standardized for primers with a Tm well below 60 °C. Homo sapiens *LRG1* gene expression [Homo sapiens leucine-rich alpha-2-glycoprotein 1 (LRG1), mRNA; NCBI reference sequence: NM_052972.3] was measured by real-time qRT-PCR using the sense 5′-TTG-GGG-AGA-ACC-AGT-TG-3′ and antisense 5′-TTC-TGG-TCA-CAG-ATC-CAG-G-3′ primers, which were generated using IDT (Integrated DNA Technologies, Coralville, IO, USA) qPCR Assay Design tools [95]. Homo sapiens *ACTB* gene expression [Homo sapiens actin beta (ACTB), transcript variant 1, mRNA; NCBI reference sequence: NM_001101] was used as a control using sense 5′-GAC-GAC-ATG-GAG-AAA-ATC-TG-3′ and antisense 5′-GAT-CTT-CAT-GAG-GTA-GTC-AG-3′ primers which were generated using IDT software [95]. The reaction was incubated in a 96-well plate at 95 °C for 2 min for PCR initial heat activation, 95 °C for 5 s for denaturation and 60 °C for 10 s for combined annealing/extension in 2-step cycling for 40 cycles, using a Rotor Gene Q real-time PCR thermal cycler instrument (Qiagen, Hilden, Germany). The qRT-PCR reaction was performed by incubation with 50 ng of RNA, 5 µL of 2× Sybr green PCR master mix, 0.1 µL of maxima Sybr green Rox reference dye, and 10 pM of sense and antisense primers, in a one-step 10 µL volume reaction. For all PCR reactions, we performed a melting curve analysis to verify the specificity and identity of PCR products. The Ct values were analyzed to determine the statistical significance of LRG1 gene expression in MDA-MB-231 cells. Relative expressions were calculated using the 2^−∆∆Ct^ method and normalized to the expression of ACTB to LRG1 [96].

To validate the data obtained from the transcriptomic microarray, ten genes with the highest differential expression were selected to evaluate their expression by real-time qRT-PCR. Homo sapiens *PIK3CD* gene expression [Homo sapiens phosphatidylinositol-4,5-bisphosphate 3-kinase catalytic subunit delta (*PIK3CD*), transcript variant 2, mRNA; NCBI: reference sequence: NM_001350234.2] was analyzed using the sense 5′-CAG-ATG-CTC-TAC-CTG-CTG-TG-3′ and antisense 5′-GTC-CAG-CAG-GAA-TTT-GGT-CA-3′ primers. Homo sapiens *HRAS* gene expression [Homo sapiens *HRas* proto-oncogene, GTPase (HRAS), transcript variant 2, mRNA; NCBI reference sequence: NM_176795.5] was analyzed using the sense 5′-GGA-TTC-CTA-CCG-GAA-GCA-GG-3′ and antisense 5′-GTC-ATC-CGA-GTC-CTT-CAC-CC-3′ primers. Homo sapiens *FGF8* gene expression [Homo sapiens fibroblast growth factor 8 (FGF8), transcript variant F, mRNA; NCBI reference sequence NM_033163.5] was analyzed using the sense 5′-GTC-TTC-ACG-GAG-ATT-GTG-CT-3′ and antisense 5′-GGG-TAG-TTG-AGG-AAC-TCG-AAG-3′ primers. Homo sapiens *ITGA7* gene expression [Homo sapiens integrin subunit alpha 7 (*ITGA7*), transcript variant 7, mRNA; NCBI reference sequence: NM_001410977.1] was analyzed using the sense 5′-ATG-GAT-GGG-AAC-CAA-TAC-CCT-3′ and antisense 5′-TAG-GTC-CAC-ACA-GAC-CGA-GT-3′ primers. Homo sapiens *CD74* gene expression [Homo sapiens CD74 antigen transcript variant 1 mRNA; NCBI reference sequence: NM_001025159.3] was analyzed using the sense 5′-GGA-GCT-GTC-GGG-AAG-ATC-AG-3′ and antisense 5′-CTG-GTA-CAG-GAA-GTA-GGC-GG-3′ primers. Homo sapiens *VEGFA* gene expression [Homo sapiens vascular endothelial growth factor A (VEGFA), transcript variant 1, mRNA; NCBI reference: NM_001025366.3] was analyzed using the sense 5′-ATC-CAA-TCG-AGA-CCC-TGG-TG-3′ and antisense 5′-GCC-TTG-GTG-AGG-TTT-GAT-CC-3′ primers. Homo sapiens MAP3K7 gene expression [Homo sapiens mitogen-activated protein kinase kinase kinase 7 (*MAP3K7*), transcript variant A, mRNA; NCBI reference: NM_003188.4] was analyzed using the sense 5′-AGA-TGA-TCG-AAG-CCC-CTT-CC-3′ and antisense 5′-GGT-TCA-CAC-GGG-ATA-ACT-GC-3′ primers. Homo sapiens *CLU* gene expression [Homo sapiens clusterin (CLU), transcript variant 1, mRNA; NCBI reference: NM_001831.4] was analyzed using the sense 5′-TAC-CTA-CCA-CTA CCT-GCC-CT-3′ and antisense 5′-TCG-TCG-CCT-TCT-CGT-ATG-AA-3′ primers. Homo sapiens *HSD17B* gene expression [Homo sapiens hydroxysteroid 17-beta dehydrogenase 2 (HSD17B2), mRNA; NCBI reference: NM_002153.3] was analyzed using the sense 5′-AGG-CTG-GCA-TCT-TAT-GGC-TC-3′ and antisense 5′-GCC-GTA-GCT-TTC-CTG-TAC-CT-3′ primers. Finally, homo sapiens *ETV7* gene expression [Homo sapiens ETS variant transcription factor 7 (ETV7), transcript variant 8, mRNA; NCBI reference: NM_001207041.2] was analyzed using 5′-CCC-GAT-ATG-AGC-CCT-ACA-TCA-3′ and antisense 5′-TCT-GAA-CAG-GAG-TTT-CTG-CCC-3′ primers. The conditions and analysis of qRT-PCR assays were the same as those described previously. All qRT-PCR assays were performed in triplicate.

### 4.11. Statistical Analysis

All experiments were conducted with a minimum of three independent biological replicates to ensure the reliability and reproducibility of the findings. The quantitative results are uniformly presented as the mean ± standard deviation. The acquired data were analyzed using established parametric statistical methods to determine significance. For qRT-PCR, the expression levels were analyzed with the 2^−ΔΔCt^ method, and statistical analysis was performed with ΔCt values using unpaired Student T tests with Welch correction to compare untreated and treated cells. Analysis of Variance (ANOVA) was utilized for comparisons involving three or more groups or complex experimental designs. All statistical computations were executed using GraphPad Prism software (version 9.0). A value of *p* < 0.05 was set as statistically significant.

## 5. Conclusions

Our findings showed that LRG1 plays a critical role in the regulation of processes associated with tumor progression in breast cancer, such as proliferation, migration, and cell survival, as assessed in the MDA-MB-231 cell line. Throughout transcriptomic and cellular enrichment analysis, we identified that LRG1 can activate key hub DEGs involved in signaling pathways participating in cancer progression, including PI3K, MAPK, and RAS. Indeed, these pathways are involved in processes such as proliferation, angiogenesis, apoptosis, and regulation of the tumor microenvironment. The results reported herein support previous studies linking LRG1 to tumor progression, highlighting its relevance as a potential therapeutic target.

## Data Availability

The datasets supporting the conclusions of this article are available in the [n9.cL] repository GSE327047, [unique persistent identifier and hyperlink to datasets: https://n9.cl/t9tg4t (accessed on 31 March 2026)]. Furthermore, we used microarray platform HCa-05-08 chip library SAGE (MWG Biotech AG., Ebersberg, Germany). The raw hybridization intensity data was found using the GenArise R package version 1.86.0 software using default parameters established in the developer manual (https://bioconductor.org/packages/genArise (accessed on 6 Febrary 2026)).

## Abbreviations

The following abbreviations are used in this manuscript:

ACTB	Actin beta
AKT	Akt Serine/threonine kinase
ANGPT2	Angiopoietin 2
ANGPTL4	Angiopoietin-like 4
ATCC	American Type Culture Collection
BAD	BCL2-associated agonist of cell death
BCL2	Oncogene B-cell leukemia 2
BDNF	Brain-derived neurotrophic factor
CASP8	Caspase 8
CDK11	Cyclin-dependent kinase 11
CD74	Cluster of Differentiation 74
CLU	Clusterin
DEGs	Differentially expressed genes
DMSO	Dimethyl sulfoxide
DUSP1	Dual-Specificity phosphatase 1
ER	Estrogen receptor alpha
EMT	Epithelial–mesenchymal transition
ECM	Extracellular matrix
ETV	ETS Variant Transcription factor
ETV7	ETS Variant Transcription factor 7
ERK	Extracellular signal-regulated kinase
FGF3	Fibroblast growth factor 3
FGFR3	Fibroblast growth factor receptor 3
FGF8	Fibroblast growth factor 8
FGF20	Fibroblast growth factor 20
FAK	Focal adhesion kinase
FBS	Fetal bovine serum
FDR	False discovery rate
GSTM1	Glutathione S-transferase Mu 1
GSTM5	Glutathione S-transferase Mu 5
GATA 1	GATA-binding protein 1
GO	Gene Ontology
HER2	Human epidermal growth factor receptor 2
HRAS	Harvey rat sarcoma viral oncogene homolog
HIF1-α	Hypoxia inducible factor 1 alpha
HK2	Hexokinase 2
HMG-CoA	3-hydroxy-3-methyl-glutaryl-coenzyme A
HSD17B	Hydroxysteroid 17β-dehydrogenase
HLA	Human Leukocyte Antigen
IGF2	Insulin-like growth factor 2
ITGB4	Integrin beta 4
ITGA7	Integrin alpha 7
IL-1	Interleukin 1
IL-6	Interleukin 6
IL-22	Interleukin 22
IRG	Immunoresponsive gene
IFNA21	Interferon alpha 21
JAK	Janus Kinase
KEGG	Kyoto Encyclopedia of Genes and Genomes
LAMB1	Laminin beta 1
LRG1	Leucine-rich alpha-2-glycoprotein 1
MAPK	Mitogen activated protein kinase
MAP2K1	Mitogen activated protein 2 kinase 1
MAP3KA7	Mitogen activated protein 3 kinase 7
MCL1	Myeloid cell leukemia sequence 1
MTT	(3-(4,5-dimethylthiazol-2-yl)-2,5-diphenyltetrazolium bromide)
MTORC1	Mechanistic Target Of Rapamycin complex 1
MDA-MB-231	MD Anderson-Metastatic Breast-231 cell line
NRAS	Neuroblastoma ras viral oncogene homolog
NF-Kβ	Nuclear factor kappa-β
NCBI	National Center for Biotechnology Information
NGF	Nerve growth factor
NT-3	Neurotrophin 3
NT-4	Neurotrophin 4
NT-5	Neurotrophin 5
PI3K	Phosphatydilinositol 3-kinase
PGF	Placental growth factor
PR	Progesterone receptor
PCR	Polymerase chain reaction
PDPK1	3-phosphoinositide-dependent protein kinase 1
PDL1	Programmed cell death ligand 1
PDGF-R	Platelet-derived growth factor receptor
PDGF	Platelet-derived growth factor receptor
RPS6	Ribosomal protein S6
RELA	RELA Protooncogene
RUNX1	Runt-related transcription factor 1
rhLRG1	Recombinant human leucine-rich alpha-2-glycoprotein 1
STAT3	Signal transducer and activator of transcription 3
STAT5B	Signal transducer and activator of transcription 5B
SMAD1	Smad family member 1
SMAD2	Smad family member 2
SMAD5	Smad family member 5
SMAD8	Smad family member 8
S6K	Ribosomal protein S6 kinase
TNFα	Tumor necrosis factor alpha
TGFβ1	Transforming growth factor beta-1
TGFβ2	Transforming growth factor beta-2
TNBC	Triple-Negative Breast Cancer
TSC1	Tuberous sclerosis complex 1
TSC2	Tuberous sclerosis complex 2
TCF1	T cell-specific 1
TBC1D7	TBC1 domain family member 7
VEGF-A	Vascular endothelial growth factor A
